# Enterogenic bacterial peritonitis and delayed chylous ascites associated with overheated peritoneal dialysis fluid infusion

**DOI:** 10.1093/ckj/sfae219

**Published:** 2024-07-13

**Authors:** Yan Sun, Cui Wang, Leping Shao

**Affiliations:** Department of Nephrology, Qingdao Municipal Hospital, Qingdao, China; Department of Nephrology, School of Medicine, the First Affiliated Hospital of Xiamen University, Xiamen University, Xiamen, China; Department of Nephrology, Beijing Tiantan Hospital, Capital Medical University, Beijing, China; Department of Nephrology, School of Medicine, the First Affiliated Hospital of Xiamen University, Xiamen University, Xiamen, China

**Figure 1:**
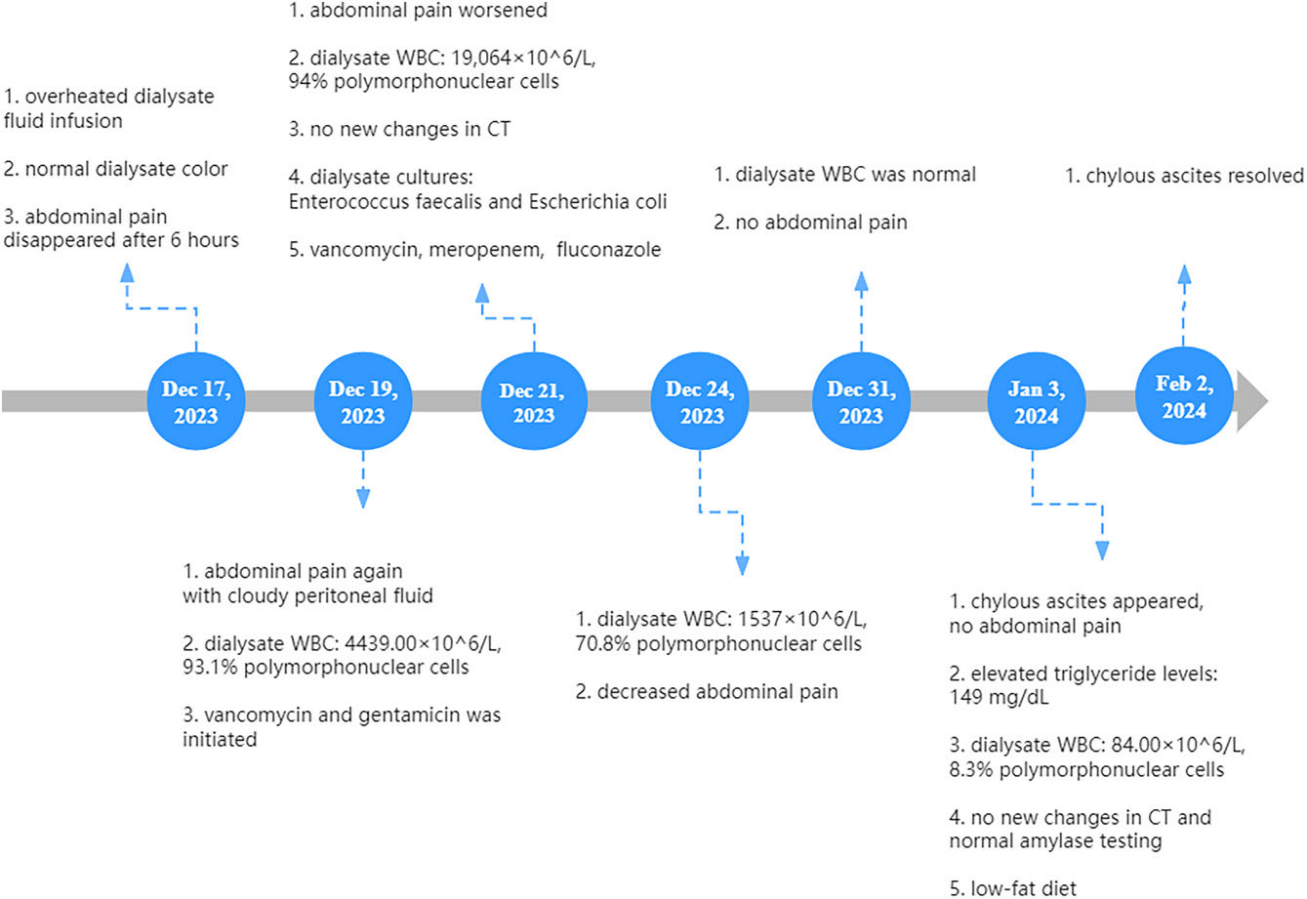
The diagnostic work up and treatment until remission.

To the Editor:

On 17 December 2023, a 78-year-old female patient undergoing continuous ambulatory peritoneal dialysis presented with abdominal pain following the infusion of ∼800 ml of overheated peritoneal dialysate (∼55°C). Physical examination revealed diffuse abdominal tenderness. Investigation by our staff revealed that the dialysate had been overheated to 55°C due to a malfunction of the temperature control device in the incubator. The volume of instilled dialysate was estimated at 800 ml by weighing the remaining dialysate in the bag. On realization of the issue, the overheated dialysate was promptly drained (∼20 minutes from the initiation of dialysate infusion to complete drainage). Subsequent instillation and drainage of peritoneal dialysis fluid at 24°C showed white flocculent material in the drained fluid, with normal color of the dialysate. The patient's abdominal pain gradually improved and subsequently disappeared after 6 hours. However, 2 days later (19 December 2023), turbid peritoneal fluid with abdominal pain and an increased leukocyte count (4439.00 × 10^6^/l, 93.1% polymorphonuclear cells) were observed. Bacterial cultures and drug sensitivity tests were conducted routinely, while empirical antimicrobial therapy with vancomycin and gentamicin was simultaneously initiated. Two days later (21 December 2023), the patient's abdominal pain worsened, accompanied by a fever (39.4°C) and an elevated CRP level (110 mg/l). Vital signs revealed a heart rate of 79 beats per minute (bpm), respiratory rate of 18 bpm, and blood pressure of 154/99 mmHg. The leukocyte count in the peritoneal fluid was 19 064 × 10^6^/l, with blood neutrophils at 10.7 × 10^6^/l. The blood culture was negative. There were no signs of consciousness disturbance or other organ failure, and abdominal CT showed no abnormalities. Positive cultures of *Enterococcus faecalis* and *Escherichia coli* prompted antibiotic adjustment, namely a switch to vancomycin and meropenem, as well as fluconazole for antifungal prophylaxis. This patient received two doses of tramadol to alleviate abdominal pain. Five days later (24 December 2023), the leukocyte count in the peritoneal fluid dropped to 1537 × 10^6^/l (70.8% polymorphonuclear cells), and abdominal pain gradually decreased, indicating a delayed response in the management of peritonitis. Immediate catheter removal may not be necessary as per ISPD recommendations. On 31 December 2023, the leukocyte count in the peritoneal fluid returned to normal. However, on 3 January 2024, the ‘milky’ chylous ascites with an elevated triglyceride level of 149 mg/dl were observed. The leukocyte count appeared normal (84.00 × 10^6^/l, 8.3% polymorphonuclear cells). The patient did not report abdominal pain or fever. Repeated abdominal CT appeared no abnormalities, and the ascitic fluid amylase level (19 U/l) ruled out the possibility of pancreatitis. The patient did not take calcium channel blockers, and the possibilities of tumors and cirrhosis were ruled out. Furthermore, negative comprehensive microbiological examinations, including staining and culture for acid-fast bacilli and sputum smear microscopy, effectively excluded tuberculosis. The patient followed a low-fat diet, and the chylous ascites resolved 4 weeks later (2 February 2024), and no further episode occurred to date. The diagnostic work up and treatment are shown in Fig. 1.

Enterogenic bacterial peritoneal infection and chylous ascites due to overheated peritoneal dialysate have not been previously reported. Some studies suggested that thermal procedures such as laparoscopic surgery or radiofrequency ablation can lead to abdominal cavity overheating, resulting in tissue damage, inflammation, and potentially severe complications such as intestinal perforation [1]. Intestinal high sensitivity to transient heat exposure at temperatures >45°C may result in intestinal barrier dysfunction, leading to bacterial translocation and peritonitis [2], allowing for the persistent diffusion of intestinal flora into the peritoneal cavity. Here, intestinal lymphatic circulation and apoptosis of lymphocytes of Peyer's patches may also play important roles for microbial translocation after thermal injury [3, 4]. These factors may have contributed to the delayed response in the management of peritonitis for this patient. While there was a timely relation between abdominal overheating, peritonitis, and chylous ascites, a causal relationship could not be confirmed. Studies have indicated that calcium channel blockers may cause chylous ascites [5], but this patient did not receive these medications, thereby excluding this possibility. We suppose that chylous ascites in this patient might related to the lymphatic tissue damage and lymphatic circulation disorders caused by thermal injury and persistent inflammatory activation due to peritonitis. Furthermore, the relatively low concentration of triglycerides could be attributed to the dilution of ascitic triglyceride concentrations by peritoneal dialysate in PD patients [5].
